# Nephroprotective potential of *Polyalthia longifolia* roots against vancomycin-induced renal toxicity in experimental animals

**DOI:** 10.3389/fphar.2023.1107435

**Published:** 2023-01-23

**Authors:** Kuntal Das, A. Muthukumar, Mansour Almuqbil, Mohd. Imran, Ali A. Rabaan, Muhammad A. Halwani, Mohammed Garout, Abdulmonem A. Alsaleh, Mohammed Alissa, Ameen S. S. Alwashmi, Ahmad A. Alshehri, Ahmed Alsayyah, Keserla Bhavani, Swati Mittal, R. Gayathri, Nasser Fawzan Alomar, Syed Imam Rabbani, Syed Mohammed Basheeruddin Asdaq

**Affiliations:** ^1^ Nitte College of Pharmaceutical Science, Yelahanka, Bangalore, India; ^2^ Central Animal House, Department of Pharmacology, Al-Ameen College of Pharmacy, Bangalore, India; ^3^ Department of Clinical Pharmacy, College of Pharmacy, King Saud University, Riyadh, Saudi Arabia; ^4^ Department of Pharmaceutical Chemistry, Faculty of Pharmacy, Northern Border University, Rafha, Saudi Arabia; ^5^ Molecular Diagnostic Laboratory, Johns Hopkins Aramco Healthcare, Dhahran, Saudi Arabia; ^6^ College of Medicine, Alfaisal University, Riyadh, Saudi Arabia; ^7^ Department of Public Health and Nutrition, The University of Haripur, Haripur, Pakistan; ^8^ Department of Medical Microbiology, Faculty of Medicine, Al Baha University, Al Baha, Saudi Arabia; ^9^ Department of Community Medicine and Healthcare for Pilgrims, Faculty of Medicine, Umm Al-Qura University, Makkah, Saudi Arabia; ^10^ Clinical Laboratory Science Department, Mohammed Al-Mana College for Medical Sciences, Dammam, Saudi Arabia; ^11^ Department of Medical Laboratory Sciences, College of Applied Medical Sciences, Prince Sattam bin Abdulaziz University, Al-Kharj, Saudi Arabia; ^12^ Department of Medical Laboratories, College of Applied Medical Sciences, Qassim University, Buraydah, Saudi Arabia; ^13^ Department of Clinical Laboratory Sciences, College of Applied Medical Sciences, Najran University, Najran, Saudi Arabia; ^14^ Department of Pathology, College of Medicine, Imam Abdulrahman Bin Faisal University, Dammam, Saudi Arabia; ^15^ Oxford College of Pharmacy, Bangalore, India; ^16^ Department of Pharmaceutics, KMCH College of Pharmacy, Coimbatore, India; ^17^ Equame Scientific and Research Center, Riyadh, Saudi Arabia; ^18^ Department of Pharmacology and Toxicology, College of Pharmacy, Qassim University, Buraydah, Saudi Arabia; ^19^ Department of Pharmacy Practice, College of Pharmacy, AlMaarefa University, Riyadh, Saudi Arabia

**Keywords:** antioxidants, antibiotics, biomarkers, vancomycin, kidney disease, treatment, Polyalthia longifolia, nephrotoxicity

## Abstract

This study was done to investigate the possible nephroprotective effect of an ethanolic root extract of *Polyalthia Longifolia* (PL) on vancomycin-induced nephrotoxicity using curative and protective models. Vancomycin (150 mg/kg, intravenous) was given to healthy Wistar albino rats in the curative model before the start of treatment, whereas the protective group received vancomycin at the conclusion of the 10-day treatment procedure. Animals were divided into six groups for both models; group I served as the normal control, while groups II, III, IV, V, and VI were kept as toxic control, standard (selenium, 6 mg/kg), LDPL (low dose of PL 200 mg/kg), HDPL (high dose of PL 400 mg/kg), and HDPL + selenium (interactive) groups, respectively. Renal biomarkers [(uric acid, creatinine, blood urea nitrogen (BUN), serum proteins], and blood electrolyte levels were measured for all tested groups. When compared to the vancomycin group, the HDPL significantly (*p* < 0.01) showed greater effectiveness in lowering the BUN, potassium, and calcium levels. Additionally, in the curative model, there was a significant (*p* < 0.05) decrease in the blood levels of uric acid, creatinine, BUN, potassium, and calcium in the animals who received the combination of selenium and HDPL. Both LDPL and HDPL did not provide any distinguishable effect in the protective model, but groups that received HDPL with selenium did provide detectable protection by significantly lowering their levels of uric acid, BUN, serum potassium, and total serum protein in comparison to the vancomycin control group. These findings indicate that, whether administered before or after renal damage is induced, the *Polyalthia longifolia* root extract provided only modest protection to nephrons, which require selenium support to prevent vancomycin-induced kidney damage.

## Introduction

Drug-induced nephrotoxicity is a prominent cause of renal failure with potentially lethal effects, and it can impede pharmaceutical research and development (Elyasi et al., 2012; Kim and Moon, 2012). It underlines the importance of developing new approaches for early and more exact diagnosis to avoid drug-induced kidney injury (Radi, 2019). Vancomycin is one such antibiotic that is most frequently used in the treatment of infectious diseases but still can cause renal damage in patients. The drug is popular due to its bactericidal properties and relatively low cost. Vancomycin is reported to be a gold standard for the treatment of methicillin-resistant *Staphylococcus aureus* (MRSA) infection (Álvarez et al., 2016). The drug was found to have an anti-bacterial spectrum against *Streptococcus sp., Enterococcus sp., Actinomyces sp., Clostridium sp.,* and *Eubacterium sp*. The mechanism of action of vancomycin was identified to involve the inhibition of cross-linking of the peptidoglycan wall of the bacteria by binding to acyl-D-Ala-D-Ala terminal (Brown et al., 2019).

Vancomycin therapy failures in individuals with methicillin-resistant *Staphylococcus aureus* infections have resulted in larger vancomycin doses to meet the required trough concentrations of 10–20 g/ML (Stogios and Savchenko, 2020). Many concerns have been raised about the safety of such high vancomycin dosages, particularly their nephrotoxicity (Perazella, 2019). Vancomycin-induced renal damage has been documented in 10%–20% and 30%–40% of individuals, respectively. Its nephrotoxicity may be exacerbated by increased reactive oxygen species and oxidative stress (Filippone et al., 2017). High trough vancomycin levels (particularly >20 mg/L) or doses (>4 g/day), concomitant nephrotoxic agent treatment, prolonged therapy (even more than 7 days), ICU admission and underlying diseased states are all considered risk factors that might accelerate or worsen vancomycin-induced nephrotoxicity (Tantranont et al., 2021).

Since ancient times, natural resources such as plants played a vital role in human race sustainability on this Earth. Plants have provided oxygen, essential nutrients, and medicines to treat human diseases. Although, human race has achieved several technological advancements but still around 80% of the world population depends on plant resources for their primary healthcare needs (Ozaslan and Oguzkan, 2018). Polyalthia is derived from ‘Greek’ which means a cure for many diseases. *Polyalthia longifolia* (PL) belonging to the family *Annonaceae* is native to many countries in the Asian continent. PL is a tree that grows up to a height of 30 feet with weeping pendulous branches and long narrow lanceolate undulate margin leaves (Yao et al., 2019). The plant is reported to have several medicinal properties and the herbal preparation were used for duodenal ulcers, fever, skin diseases, diabetes, hypertension, helminthiasis, hepato-protective, antibacterial, anti-inflammatory, and anti-cancer (Chanda et al., 2011; Vijayarathna et al., 2017; Rai et al., 2021). The extracts of PL were reported to possess antiviral property (Yadav et al., 2020). Administration of PL was found to produce synergistic activity with antibiotics against methicillin-resistant *Staphylococcus aureus* (Kirubakari et al., 2020). Besides, two clerodane diterpenes isolated from PL were reported to exhibit anti-histaminic and anti-helicobacter pylori activities (Edmond et al., 2021). Furthermore, apoptosis-inducing properties essential for the eradication of tumors has been found in the extracts of PL (Afolabi et al., 2019). PL is reported to contain diterpenoids, alkaloids, tannins, and mucilage (Lee et al., 2009). Some of the important active ingredients identified were O-methyl bulbocapnin-N-oxide, N-methyl nandigerine-N-oxide, Oliver online-N-oxide, pendulamine A, 8-oxopolyalthiane and 13-halimadien-15-oic acid. These components have been linked to the reported pharmacological activities of the plant (Lee et al., 2009; Bhattacharya et al., 2015).

Therefore, this study was designed to investigate the protective and curative potential of *Polyalthia longifolia* against vancomycin-induced nephrotoxicity using biochemical markers (serum creatinine, BUN, serum uric acid, and total serum protein), and serum electrolytes (sodium, potassium, chloride, and calcium) measurements.

## Materials and methods

### Drugs, chemicals, and biomedical reagents

The chemicals and reagents used in the study were of analytical grade meant for research purposes. These were procured from the regular chemical supplier of the institution by placing an order in the central storehouse. Vancomycin was obtained from Cipla Limited, Bangalore, India, and diagnostic equipment from Oscar Medicare Private Limited, Bangalore, India.

### Root extraction

In January 2021, matured and fresh roots were obtained from Lalbagh botanical garden in Bangalore, Karnataka, India. Dr. V. Rama Rao, Research Officer (S-2) Botany, and Dr. Sulochana Bhat, Assistant Director (S-4) In-charge, from the Central Ayurveda Research Institute, Bangalore, authenticated the plant roots, which were then deposited in the al-Ameen central herbarium. (Authentication/SMPU/CARI/BNG/2021-22/35). 3kg of adult *Polyalthia longifolia* leaves were collected, dried, and powdered. For 72 h, the powdered (1,200 g) roots were macerated in 80% ethanol. The final leaf extract was filtered through Whatman filter paper and lyophilized under reduced pressure. The yield percentage obtained was 31.12% w/w. The ethanolic root extract was then subjected to additional pharmacological testing.

### Selection of experimental animals

Six to 7 weeks old albino Wistar rats weighing 150–180 gm (equal number of male and female rats) was obtained from a central animal house at the Al-Ameen College of Pharmacy in Bangalore and kept in an animal room under standard environmental conditions (50% relative humidity, a light-dark cycle of up to 12 h, and a room temperature of 28°C). They were also fed a standard diet and given access to water at will. This investigation was confirmed by the institutional animal ethics committee (IAEC) and given their support. The normal rules and policies of CPCSEA were adhered while conducting experiments with testing animals and providing them with particular care (Reg. AACP/IAEC/JAN2020/10).

### Protocol design

#### Method for the induction

The test drug as fine powder was suspended in 0.5% aqueous carboxyl methylcellulose (CMC) suspension. Every day, the test dose suspension was freshly prepared. A feeding cannula was used to administer the homogenized suspension, which had been thoroughly shaken for 1 min. The same method was used to make the daily new suspension. The animals were divided into six groups of six animals each at random. Vancomycin was given intravenously at a dose of 150 mg/kg body weight (Pais et al., 2020). The doses of *Polyalthia longifolia* for curative and protective models were selected based on the research reported in the literature (Oyeyemi et al., 2020).

#### Curative model protocol (Gao et al., 2020)

Group 1 (Normal control) animals were given a normal saline injection intraperitoneal on first day, and 0.3% CMC aqueous solution was given orally on the 6th day and continued for 10 days. Animals in Group 2 (Positive control) were given vancomycin 150 mg/kg i.v. on the first day, and 0.3% CMC aqueous solution was given orally on the 6th day for 10 days. Animals in Group 3 were given vancomycin 150 mg/kg i.v on the first day; however, starting on the 6th day, the standard drug, selenium, was also given in the dose of 6 mg/kg body weight once daily orally for the next 10 days. Animals in Groups 4 and 5 were given vancomycin 150 mg/kg i.v. on the first day; however, starting on the 6th day, lower (200 mg/kg) and higher (400 mg/kg) of PL, respectively (Oyeyemi et al., 2020), were also given orally once daily for the next 10 days. On the first day, Group 6 received vancomycin 150 mg/kg i.v; however, beginning on the 6th day a combination of selenium (6 mg/kg) with PL (400 mg/kg) was administered orally, daily for the next 10 days. Animals were fasted overnight with free access to drinking water and the sampling for biochemical estimation was done after 24 h of last dose of the treatment.

#### Protective model protocol (Liu et al., 2020)

Group 1 (Normal control): Animals were given 0.3% CMC aqueous solution orally for 10 days before receiving an i.v injection of normal saline on the 11th day. Group 2 (Positive control): Animals were given 0.3% CMC aqueous solution orally for 10 days, followed by 150 mg/kg i.v vancomycin on the 11th day. Animals in Group 3 received standard drug selenium at a dose of 6 mg/kg body weight orally once daily for 10 days, and vancomycin injection was given intravenously on the 11th day at a dose of 150 mg/kg bw. Animals in Group 4 received test drug PL at a dose of 200 mg/kg body weight (Oyeyemi et al., 2020) orally once daily for 10 days, and vancomycin injection was given intravenously on the 11th day at a dose of 150 mg/kg bw. Animals in Group 5 received test drug PL at a dose of 400 mg/kg body weight orally once daily for 10 days, and vancomycin injection was given intravenously on the 11th day at a dose of 150 mg/kg bw. Group-6 animals received standard drug selenium (6 mg/kg) and test drug PL (400 mg/kg, b.w) orally, once daily for 10 days, and vancomycin injection was given intravenously on the 11th day in the dose of 150 mg/kg bw. Animals were fasted overnight with free access to drinking water and the sampling for biochemical estimation was done after 24 h of vancomycin injection.

### Biochemical assay

At the end of the experiment, the rats were anaesthetized for 50–60 s with isoflurane (EZ anaesthesia system), and 2–3 mL of blood was collected from the retro-orbital plexus for biochemical analysis. The biochemical parameters such as uric acid, creatinine, BUN, and total proteins were estimated using the diagnostic kits supplied by Oscar Medicare Pvt Ltd. In addition, blood electrolytes levels (sodium, potassium, chloride, and calcium) were also measured using the Oscar Medicare diagnostic kits. The procedure described in each user’s manual of the kit was followed while estimating the biomarker parameters (URIC-ACID, 2020; Al-Doaiss et al., 2021). Samples were obtained from each animal of all groups (in multiple of six for each group) and coded for blinding.

### Statistical investigation

For statistical purposes, GraphPad Prism Version 5.0 software ran a one-way analysis of variance (ANOVA) followed by Tukey’s test on all the raw data collected during the study. Sample size of animals was calculated from the formula proposed in the literature. The degree of freedom, confidence interval (95%), repeat administration, and the number of groups in the study was considered while deciding the total number of rats needed (Arifin and Zahiruddin, 2017). Based on the calculation, 7-8 animals were included in each group and comprised of animals of both sexes. The values were given as mean SEM, and a *p-value* of 0.05 was statistically significant.

## Results

### Curative effect of PL and selenium against vancomycin-induced nephrotoxicity

#### Renal biomarkers

Comparison between the baseline values of uric acid, creatinine, BUN and total proteins with normal control indicated non-significant variation. Further, the observations indicated that administration of vancomcycin (150 mg/kg) non-significantly increased the uric acid levels compared to control group. Treatments such as selenium (6 mg/kg), low dose of PL (200 mg/kg) and high dose of PL (400 mg/kg) after vancomycin did no induced significant variation in uric acid levels. However, when selenium (6 mg/kg) and PL (400 mg/kg) was tested in combination, a significant (*p* < 0.05) reduction in uric acid level was observed compared to positive control group. On the other hand, administration of vancomycin (150 mg/kg) showed significant (*p* < 0.05) increase in creatinine levels compared to normal control group. Treatment of selenium (6 mg/kg), lower dose of PL (200 mg/kg) and higher dose of PL (400 mg/kg) did not produce any significant change in the creatinine level. Further, the combination of selenium (6 mg/kg) and PL (400 mg/kg) produced a significant (*p* < 0.01) reduction in creatinine level compared to positive control group. Similar observation was found for BUN, where vancomycin (150 mg/kg) significantly (*p* < 0.05) elevated its level and diminishing action was found only with combination of selenium and PL at high dose was tested. Moreover, none of the treatments significantly altered the total protein levels (Table 1).

**TABLE 1:** Effect of curative administration of PL and selenium on the renal biomarkers in rats treated with vancomycin.

Renal biomarkers	Baseline values (NS, i,p)	Normal control (NS i.p + 0.3% CMC, p.o)	Positive control (150 mg/kg, i.v + 0.3% CMC, p.o)	Vanco (150 mg/kg i.v) +Standard selenium (6 mg/kg, p.o)	Vanco (150 mg/kg i.v) +PL (200 mg/kg, p.o)	Vanco (150 mg/kg i.v) +PL (400 mg/kg, p.o)	Vanco (150 mg/kg i.v) And selenium (6 mg/kg, p.o) + PL (400 mg/kg, p.o)
Uric acid (mg/dL)	1.81 ± 0.08	1.99 ± 0.10	2.66 ± 0.14	1.87 ± 0.05	1.93 ± 0.09	1.82 ± 0.03	1.72 ± 0.05*
Creatinine (mg/dL)	0.88 ± 0.04	0.82 ± 0.06	1.71 ± 0.08^a^	0.96 ± 0.08	1.28 ± 0.11	0.99 ± 0.09	0.76 ± 0.03**
BUN (mg/dL)	18.96 ± 1.06	17.62 ± 0.72	68.95 ± 1.33^a^	36.12 ± 1.07	50.57 ± 1.41	43.02 ± 1.44*	30.62 ± 1.00**
Total protein (g/dL)	5.96 ± 0.48	6.23 ± 0.17	6.87 ± 0.03	6.15 ± 0.03	6.39 ± 0.20	6.76 ± 0.14	6.69 ± 0.16

NS, Normal saline; CMC, Carboxy methylcellulose, Vanco—Vancomycin; PL, *Polyalthia longifolia*.

Values expressed as mean ± SEM; Statistics: One-way ANOVA.

^a^ p< 0.05 compared with normal control; *p < 0.05, **p < 0.01, compared with positive control.

#### Blood electrolytes

The observation of Table 2 suggests that administration of vancomycin (150 mg/kg) non-significantly increased the levels of sodium, potassium, chloride, and calcium. Treatment of selenium (6 mg/kg) did not produce significant alteration in the blood electrolyte levels. However, both lower dose (200 mg/kg) and higher dose (400 mg/kg) of PL showed significant reduction (*p* < 0.05) in the elevated potassium and calcium levels induced by vancomycin. These effects were found to become prominent (*p* < 0.001) when combination of selenium and PL (400 mg/kg) was tested in vancomycin. Moreover, no significant variation was observed when comparison was done between the baseline values and normal control values.

**TABLE 2:** Effect of curative administration of PL and selenium on blood electrolyte levels in rats treated with vancomycin.

Electrolyte levels (mmol/L)	Baseline values (NS, i,p)	Normal control (NS i.p + 0.3% CMC, p.o)	Positive control (150 mg/kg, i.v + 0.3% CMC, p.o)	Vanco (150 mg/kg i.v) +Standard selenium (6 mg/kg, p.o)	Vanco (150 mg/kg i.v) +PL (200 mg/kg, p.o)	Vanco (150 mg/kg i.v) +PL (400 mg/kg, p.o)	Vanco (150 mg/kg i.v) And selenium (6 mg/kg, p.o) + PL (400 mg/kg, p.o)
Sodium	153.21 ± 2.14	144.93 ± 0.84	155.25 ± 0.72	143.73 ± 0.48	149.12 ± 0.45	146.28 ± 0.45	142.33 ± 0.60
Potassium	6.23 ± 0.09	5.88 ± 0.23	6.30 ± 0.02	4.86 ± 0.05	5.91 ± 0.11*	5.53 ± 0.07**	4.37 ± 0.08***
Chloride	88.24 ± 0.72	94.95 ± 0.58	97.26 ± 0.39	93.04 ± 0.47	94.97 ± 0.32	92.95 ± 0.36	90.53 ± 0.39
Calcium	2.26 ± 0.39	2.64 ± 0.02	2.84 ± 0.05	2.74 ± 0.07	2.80 ± 0.05*	2.78 ± 0.11*	2.65 ± 0.02***

NS, Normal saline; CMC, Carboxy methylcellulose, Vanco—Vancomycin; PL, *Polyalthia longifolia*.

Values expressed as mean ± SEM, Statistics: One-way ANOVA; *p < 0.05, **p < 0.01, ***p < 0.001 compared with positive control.

### Protective effect of PL and selenium in vancomycin induced nephrotoxicity

#### Renal biomarkers

The observations from Table 3 suggest that vancomycin (150 mg/kg) treatment towards end of the study did not induce significant variation in uric acid level compared to normal control group. Similarly, selenium (6 mg/kg), PL (200 mg/kg) and PL (400 mg/kg) treatment also did not alter the uric acid level significantly. However, combination of selenium (6 mg/kg) and PL (400 mg/kg) produced a significant (*p* < 0.01) reduction in uric acid level compared to positive control group. The estimation of creatinine level indicated that vancomycin (150 mg/kg) significantly (*p* < 0.05) increased it compared to normal control. Individual treatments of selenium (6 mg/kg), PL (200 mg/kg) and PL (400 mg/kg) did not affect the creatinine levels in vancomycin administered animals. However, combination of selenium (6 mg/kg) and PL (400 mg/kg) reduced significantly (*p* < 0.05) the blood creatinine levels compared to vancomycin group. Similar observations were recorded for BUN estimation, wherein vancomycin increased its level significantly (*p* < 0.01) compared to control group and none of the treated groups except combination of selenium (6 mg/kg) and PL (400 mg/kg) was found to be effective in reducing the BUN levels. The combined drug treatment reduced the BUN level significantly (*p* < 0.05) compared to vancomycin treated animals. The estimation of total protein levels indicated that the combination of selenium and PL significantly (*p* < 0.05) enhanced the level compared to positive control group, while other treatments did not induce any significant variation. No significant variation was observed when baseline values were compared with the normal control group.

**TABLE 3:** Effect of preventive administration of PL and selenium on renal biomarkers in vancomycin treated rats.

Renal biomarkers	Baseline values (NS, i,p)	Normal control (NS i.p + 0.3% CMC, p.o)	Positive control (150 mg/kg, i.v + 0.3% CMC, p.o)	Vanco (150 mg/kg i.v) +Standard selenium (6 mg/kg, p.o)	Vanco (150 mg/kg i.v) +PL (200 mg/kg, p.o)	Vanco (150 mg/kg i.v) +PL (400 mg/kg, p.o)	Vanco (150 mg/kg i.v) And selenium (6 mg/kg, p.o) + PL (400 mg/kg, p.o)
Uric acid (mg/dL)	1.78 ± 0.62	1.86 ± 0.04	2.66 ± 0.14	1.80 ± 0.06	1.93 ± 0.09	1.82 ± 0.03	1.69 ± 0.03**
Creatinine (mg/dL)	1.03 ± 0.11	1.15 ± 0.06	1.88 ± 0.07^a^	1.22 ± 0.08	1.60 ± 0.06	1.40 ± 0.13	1.01 ± 0.14
BUN (mg/dL)	15.82 ± 0.34	16.88 ± 0.61	28.98 ± 0.66^b^	21.24 ± 0.88	25.14 ± 0.33	22.03 ± 0.51	19.53 ± 0.48*
Total protein (g/dL)	6.79 ± 0.56	7.12 ± 0.02	6.87 ± 0.03	7.14 ± 0.01	7.21 ± 0.04	7.12 ± 0.01	7.39 ± 0.05*

NS, Normal saline; CMC, Carboxy methylcellulose, Vanco—Vancomycin; PL, *Polyalthia longifolia*.

Values expressed as mean ± SEM, Statistics: One-way ANOVA.

^a^p< 0.05.

^b^ p< 0.01 compared with normal control; *p < 0.05, **p < 0.01 compared with positive control.

#### Blood electrolytes

The influence of PL and selenium treatments on the blood electrolytes levels was recorded by estimating sodium, potassium, chloride, and calcium levels. Comparison of baseline data and normal control values revealed non-significant variation. Further, the observations indicated that none of the treatments significantly altered the blood sodium levels. Similarly, the findings of chloride and calcium estimations suggest that none of the treatments including vancomycin induced significant change in their serum levels. However, the blood potassium estimation suggests that vancomycin (150 mg/kg) significantly (*p* < 0.05) elevated its level compared to control. The combination of selenium (6 mg/kg) and PL (400 mg/kg) significantly (*p* < 0.01) reduced the elevated potassium level compared to vancomycin and none of the other treatments showed any significant variation (Table 4).

**TABLE 4:** Effect of preventive administration of PL and selenium on blood electrolyte levels in rats treated with vancomycin.

Electrolyte levels (mmol/L)	Baseline values (NS, i,p)	Normal control (NS i.p + 0.3% CMC, p.o)	Positive control (150 mg/kg, i.v + 0.3% CMC, p.o)	Vanco (150 mg/kg i.v) +Standard selenium (6 mg/kg, p.o)	Vanco (150 mg/kg i.v) +PL (200 mg/kg, p.o)	Vanco (150 mg/kg i.v) +PL (400 mg/kg, p.o)	Vanco (150 mg/kg i.v) And selenium (6 mg/kg, p.o) + PL (400 mg/kg, p.o)
Sodium	114.21 ± 1.85	125.20 ± 0.87	156.64 ± 0.65	133.37 ± 0.60	148.22 ± 0.79	138.74 ± 0.92	138.83 ± 0.73
Potassium	7.88 ± 0.46	7.01 ± 0.22	14.81 ± 0.61^a^	8.83 ± 0.16	11.71 ± 0.90	10.77 ± 0.37	9.75 ± 0.32*
Chloride	95.36 ± 1.09	105.71 ± 0.68	117.43 ± 0.39	91.25 ± 0.62	95.48 ± 0.73	92.95 ± 0.36	90.27 ± 0.63
Calcium	1.72 ± 0.55	1.64 ± 0.02	2.14 ± 0.10	1.74 ± 0.02	1.81 ± 0.01	1.73 ± 0.01	1.65 ± 0.02

NS, Normal saline; CMC, Carboxy methylcellulose, Vanco—Vancomycin; PL, *Polyalthia longifolia*.

Values expressed as mean ± SEM, Statistics: One-way ANOVA.

^a^p < 0.05 compared with normal control; *p < 0.01 compared with positive control.

## Discussion

The present study was conducted to evaluate the protective and curative potential of *Polyalthia longifolia* (PL) against vancomycin induced nephrotoxicity. Vancomycin is a complex tricyclic glycopeptide antibiotic obtained from the microorganism *Streptococcus orientalis*. The drug has erratic gastrointestinal absorption characteristics and is thus mostly administered by parenteral route such as intravenous (Álvarez et al., 2016). Vancomycin is preferred for treating severe infections caused by different strains of microorganisms including some of the resistant ones. The drug is considered an effective alternative in patients hypersensitive to penicillins and/or cephalosporins as well admitted to intensive care units for the treatment of pneumonia, empyema, endocarditis, osteomyelitis, and soft tissue abscess (Brown et al., 2019). However, vancomycin treatment is reported to be associated with several adverse effects such as hypotension, tachycardia, phlebitis, nephrotoxicity, ototoxicity, exanthema, and hypersensitivity reactions (Filippone et al., 2017).

In this study, when vancomycin was administered on the first day (curative model), it significantly (*p* < 0.05) increased the creatinine and BUN levels (Table 1) without inducing marked variation in the blood electrolytes (Table 2). Similar observations were recorded in protective strategy when vancomycin was administered to animals on 11th day (Table 3). However, in this model, vancomycin was observed to increase the blood potassium level significantly (*p* < 0.05) compared to control group (Table 4).

The observation of this study suggested that vancomycin (150 mg/kg) has induced renal toxicity without altering the electrolyte levels, except the blood potassium levels. The findings contradict the earlier findings wherein vancomycin was reported to produce hypokalemia in patients receiving the drug. The study suggested that the infectious state of the patients could be an important factor related to vancomycin-induced hypokalemia (Karimzadeh et al., 2016). Further, the effect of vancomycin on renal parameters such as creatinine and BUN are in accordance with the previous research where vancomycin elevated these biomarkers in rats (Elaidy, 2013).

The experimental data available in the literature indicates that oxidative stress due to generation of reactive oxygen species as the major cause for nephrotoxicity. To support this, previous studies indicated that vancomycin lowered glutathione levels while increasing the lipid peroxidation in renal mitochondria (Xu et al., 2021). In kidney tissues, vancomycin was observed to inhibit antioxidant enzymes such as glutathione transferase, glutathione peroxidase and superoxide dismutase, causing cellular redox status to shift and toxic reactive oxygen species to rise (Guzel et al., 2020).

Several strategies have been attempted to improve the safety profile of vancomycin. One of the most logical approaches is to use a known antioxidant to reduce the oxidative stress-induced damages on the renal tissues (Kandemir et al., 2018). Some of the compounds derived from natural resources such as thymoquinone, curcumin and 1,5-Isoquinelinediol have been tested and were found to be efficacious in reducing the vancomycin-mediated nephrotoxicity (Dalaklioglu et al., 2010; Ahmida, 2012; Basarslan et al., 2012). Earlier studies have indicated that PL administration is well tolerated in different models of toxicity. The brine shrimp lethality assay revealed that PL possess very low level of general toxicity and the LC_50_ and LC_90_ values were observed to be 20 μg/ml and 70–80 μg/ml, respectively (Chanda et al., 2012). An *in-vivo* study conducted on Wistar rats indicated that the extract of PL did not produce any signs of toxicity when tested up to 3,240 mg/kg, b.w. The study suggested that administration of the extract for 14 days did not induce alteration in the body weight, food or water intake, organ weights, biochemical and hematology in both sexes of the animals (Jothy et al., 2013).

Administration of PL at lower dose (200 mg/kg) and higher dose (400 mg/kg) were tested for protective and curative action against vancomycin-induced renal damages. In the curative strategy, the monotherapy of PL (200 mg/kg) and selenium (6 mg/kg) to the vancomycin treated rats did not induce significant alteration in the levels of uric acid, creatinine, BUN and total proteins. Similarly, the higher dose of PL (400 mg/kg) also did not produced significant variation except for BUN, where it lowered the levels (37.6%) compared to positive control group. However, when combination of selenium (6 mg/kg) and PL (400 mg/kg) was tested in the vancomycin, a reduction was observed in the levels of uric acid (35.3%), creatinine (55.6%) and BUN (55.8%) compared to positive control group (Table 1). On the other hand, PL alone as well in combination with selenium showed a dose-dependent reduction on the levels of potassium and chloride in vancomycin-treated animals. The percentage reduction in potassium level was found to be 6.2% with PL—200 mg/kg, 12.2% with PL—400 mg/kg, 30.6% with combination, and chloride level was 1.4% with PL—200 mg/kg, 2.1% with PL—400 mg/kg, 6.6% with combination (Table 2)

The protocol to test the protective effect of test compounds indicated that the monotherapy of PL (200 and 400 mg/kg) as well as selenium (6 mg/kg) did not induced significant variation in the levels of uric acid, creatinine, BUN and total proteins compared to the positive control group. Further, when combination of higher dose of PL (400 mg/kg) and selenium (6 mg/kg) was tested in the vancomycin treated group, a reduction in the levels of uric acid (36.1%) and BUN (32.6%) and elevation of total proteins (7.4) was observed (Table 3). The monotherapy of PL and selenium did not produce significant alteration in the electrolyte levels but the combination of selenium (6 mg/kg) and PL (400 mg/kg) reduced (34.1%) the potassium level in vancomycin treated animals (Table 4).

The observations of the study indicated that PL in both the tested models did not induce significant reversal against vancomycin mediated renal changes (Table 1; Table 3). However, elevated potassium level was found to be significantly reduced in the curative strategy (Table 3). Selenium being a known antioxidant prevented the vancomycin-induced renal damages in an earlier study (Asefaw et al., 2020). However, in the present study it was used as standard and showed non-significant variation on the altered renal biomarker levels induced by vancomycin (Table 1; Table 3). The blood electrolyte levels were also found to be non-significantly influenced by selenium (Table 2; Table 4).

An important observation of the study is that when combination of selenium (6 mg/kg) and PL (400 mg/kg) was tested against vancomycin, a significant reversal of elevated uric acid, creatinine and BUN level was seen in both curative (Table 1) as well as protective models (Table 2). Uric acid is formed from metabolic breakdown of purine nucleotides, while creatinine is a waste product obtained from muscle and protein metabolism. During the urea cycle, liver produces urea as a waste substance from the protein digestion. These waste products are primarily removed by kidneys through glomerular filtration and the elevation of their levels in blood indicates the failure in the renal function (Sinha et al., 2016). Vancomycin in the earlier studies induced nephrotoxicity by affecting the glomerular filtration rate and the combination of the selenium with PL appears to have counteracted this action of vancomycin (Mehanna et al., 2022). Besides, the combination therapy was found to be effective in reducing the blood potassium levels in both curative and protective strategies (Table 2; Table 4). Earlier studies have indicated that PL possesses antioxidant property (Chen et al., 2014).

Observations of this study suggest that combination of selenium and PL might have potentiated the antioxidant activity, and this might have effectively managed the oxidative stress generated by vancomycin in minimizing the nephrotoxicity, which was earlier not achieved with monotherapy. Further, PL has been reported to possess antimicrobial property [42] and this action could play a positive role in the therapeutic efficacy of vancomycin besides minimizing the toxicity reactions. Besides, the non-significant variation in the blood electrolyte levels indicated that none of the treatments tested in the study need additional therapeutic interventions such as diuretics to maintain the electrolyte homeostasis. However, more studies are required to precisely establish either the protective or curative role of PL in vancomycin induced systemic toxicity including testing the early nephrotoxicity biomarkers such as kidney injury molecule-1 (KIM-1) and neutrophil geletinase-associated lipocalin (NGAL).

## Conclusion

It can be concluded that the nephroprotective effect of PL was correlated with its ability to improve renal function parameters. A possible mechanism of PL is increased glomerular filtration rate resulting in decreased serum creatinine while maintaining serum magnesium and potassium levels. PL’s curative and protective effect against Vancomycin toxicity may also be due to its powerful antioxidant and detoxification properties and these might have prevented the inflammatory response to maintain the normal kidney function. The precise mechanism of action may be the subject of a separate, exhaustive study. Consequently, the present study classifies PL as a possible nephroprotective agent and supports the traditional natural claim regarding its activity and use in renal disorders.

## Data Availability

The original contributions presented in the study are included in the article/supplementary material, further inquiries can be directed to the corresponding author.
